# Fungal Contamination of Methylprednisolone Causing Recurrent Lumbosacral Intradural Abscess

**DOI:** 10.3201/eid2303.161334

**Published:** 2017-03

**Authors:** Jaclyn J. Renfrow, Mark B. Frenkel, Wesley Hsu

**Affiliations:** Wake Forest Baptist Medical Center, Winston-Salem, North Carolina, USA

**Keywords:** contaminated methylprednisolone injection, epidural steroids, fungal abscess, fungi

## Abstract

Fungal meningitis transmitted through injections of methylprednisolone contaminated with *Exserohilum rostratum* affected 753 persons and caused 61 deaths in the United States in 2012. We report a case of infection recurrence after 24-months with the unique manifestation of an intradural fungal abscess. Fungal disease should remain on the differential diagnosis list for previously exposed patients.

Fungal meningitis transmitted through injections of methylprednisolone contaminated with *Exserohilum rostratum* affected 753 persons and caused 61 deaths in the United States in 2012 (*1*). More than 13,400 patients were potentially exposed to 1 of 3 contaminated drug lots (*2*,*3*). However, whether recommended treatment eliminates this disease is unknown because of limited reports of recurrent disease (*4*). 

As of December 2015, the Centers for Disease Control and Prevention (CDC) had reported 8 cases of *E. rostratum* meningitis relapses within a median time of 90 days. Reporting recurrent cases informs potential treatment recommendation changes and long-term care guidelines for affected persons. With institutional review board approval from Wake Forest University Medical Center, we describe fungal infection recurrence at 24 months.

A 78-year-old woman sought treatment at the neurosurgery clinic at Wake Forest Medical Center (Winston-Salem, NC, USA), in August 2015 with a 4-month history of acute or chronic lower back pain, leg weakness, and radicular pain in the left side. Nonoperative interventions, including narcotics, physical therapy, and epidural steroid injections (L5–S1, July 2015), did not control her symptoms.

The patient had received an injection of contaminated methylprednisolone on September 12, 2012. She was identified as a patient affected by the contamination and contacted as part of the CDC investigation. One month after contact, she was hospitalized for intractable headaches, nausea, and vomiting.). PCR results from 2 samples of cerebrospinal fluid (CSF) performed by CDC were positive for *E. rostratum* (from October 16 and 22, 2012). Treatment consisted of intravenous voriconazole, later switched to ambisome, with a transition to oral voriconazole due to hallucinations. Therapy response was monitored with serial lumbar punctures. Contrast-enhanced magnetic resonance imaging (MRI) of the lumbar region was performed on November 18, 2012, and showed an enhancing epidural abscess, spanning T12–S2. Treatment was discontinued on January 16, 2013, a decision supported by 10 serially negative CSF fungal cultures and repeat PCR, negative for *E. rostratum,* performed at CDC on February 22, 2013. Lumbar MRI on February 1, 2013, showed improvement of the lumbar epidural abscess. 

The October 2012 hospitalization was complicated by the patient’s persistent altered mental status and right hemiparesis, which prompted a contrast-enhanced MRI of the brain on February 15, 2013, that demonstrated a left transverse sinus thrombus. The condition was monitored, and repeat imaging on March 1, 2013, found it to be nearly resolved. The patient appeared to be recovered from her infection at her last infectious disease follow-up in March 2013.

The patient was hospitalized again in May 2015 after a fall; she experienced worsening back pain, headaches, and confusion. Brain MRI demonstrated a recurrent dural venous thrombosis, which was treated with anticoagulants. Given concern for recurrent fungal infection, she also underwent lumbar puncture. CSF cell count and chemistries were within normal limits. CSF cultures were negative for fungi. 

At home, her acute left leg pain worsened, leaving her nonambulatory, and she sought hospital management. On neurologic examination, she was awake and alert with some delirium/confusion and some mild weakness in the left lower leg. Contrast-enhanced lumbar MRI demonstrated a homogeneous enhancing intradural mass, spanning L4 to the sacrum, with a corresponding T2 hypointense signal (Technical Appendix Figure, panels A, B). Diagnosing this lumbosacral intradural mass was not obvious because the differential diagnosis includes neoplasms, infections, and hematomas. Given the patient’s worsening symptoms, we performed nerve decompression and resection of the mass.

During the operation, the patient underwent a laminectomy of L3–L5; intraoperative findings showed an intradural abscess and arachnoiditis, with edema and adherence of the cauda equina nerve roots (Figure). Pathologic examination demonstrated abundant necrotic material containing septate hyphae fungal elements of brown pigment, consistent with a dematiaceous fungus. The material did not undergo PCR, given her clinical history (including PCR) and pathologic findings at recurrence. Her condition was treated with intravenous amphotericin B and voriconazole during her 12-day hospitalization, and she was discharged on oral voriconazole for outpatient treatment, with an anticipated duration of 1 year. At 5-month follow-up, she had complete resolution of her back pain and was full strength with some intermittent left radicular pain.

**Figure:**
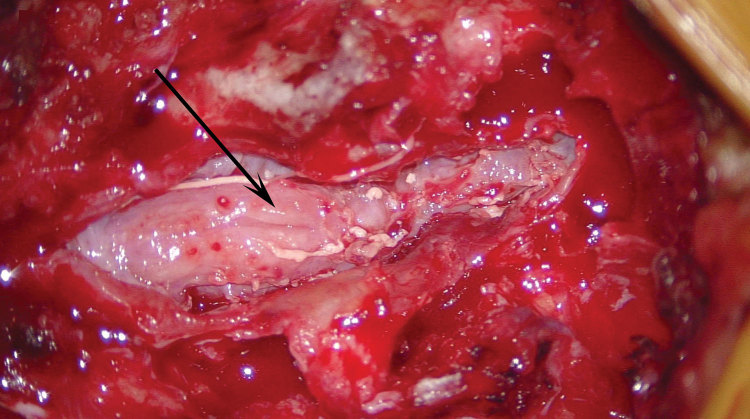
Intraoperative image demonstrating postevacuation cauda equina nerve roots that are grossly edematous and adherent (arrow), consistent with arachnoiditis, in a patient with recurrent infection from fungal-contaminated methylprednisolone, North Carolina, USA, 2015.

Only 3 other cases of intradural abscess were reported from the initial outbreak, making this recurrence a notable CNS disease manifestation (*5*). The patient had several risk factors for recurrence. She had received epidural steroid injections after antifungal treatment; the steroids resulted in an immunocompromised environment, potentially allowing for immune evasion and residual disease. A dural rent during multiple spinal taps or posttreatment epidural steroid injections may have seeded the fungus in the intradural space, which then expanded because antifungal agents demonstrate relatively poor CSF penetration. She also underwent a 3-month initial treatment; at least 6 months of antifungal treatment is currently recommended, although optimal treatment duration remains uncertain because objective criteria for infection clearance are lacking. 

Given the potential for recurrence, fungal disease should remain on the differential diagnosis list for patients with prior exposure. In addition, long-term follow-up could identify patients needing further treatment (*4*).

Technical Appendix Enhanced lumber mass in a patient with recurrent infection from fungal-contaminated methylprednisolone.
