# Supplementary material for: Fungal Contamination of Methylprednisolone Causing Recurrent Lumbosacral Intradural Abscess
Source: Emerg Infect Dis. 2017 Mar;23(3):552–3. doi: 10.3201/eid2303.161334 (PMC5382764; doi:10.3201/eid2303.161334)
Supplement: Technical Appendix — Enhanced lumber mass in a patient with recurrent infection from fungal-contaminated methylprednisolone. [file 16-1334-Techapp-s1.pdf]

Article DOI: http://dx.doi.org/10.3201/eid2303.161334

Fungal Contamination of Methylprednisolone Causing Recurrent Lumbosacral Intradural Abscess

Technical Appendix

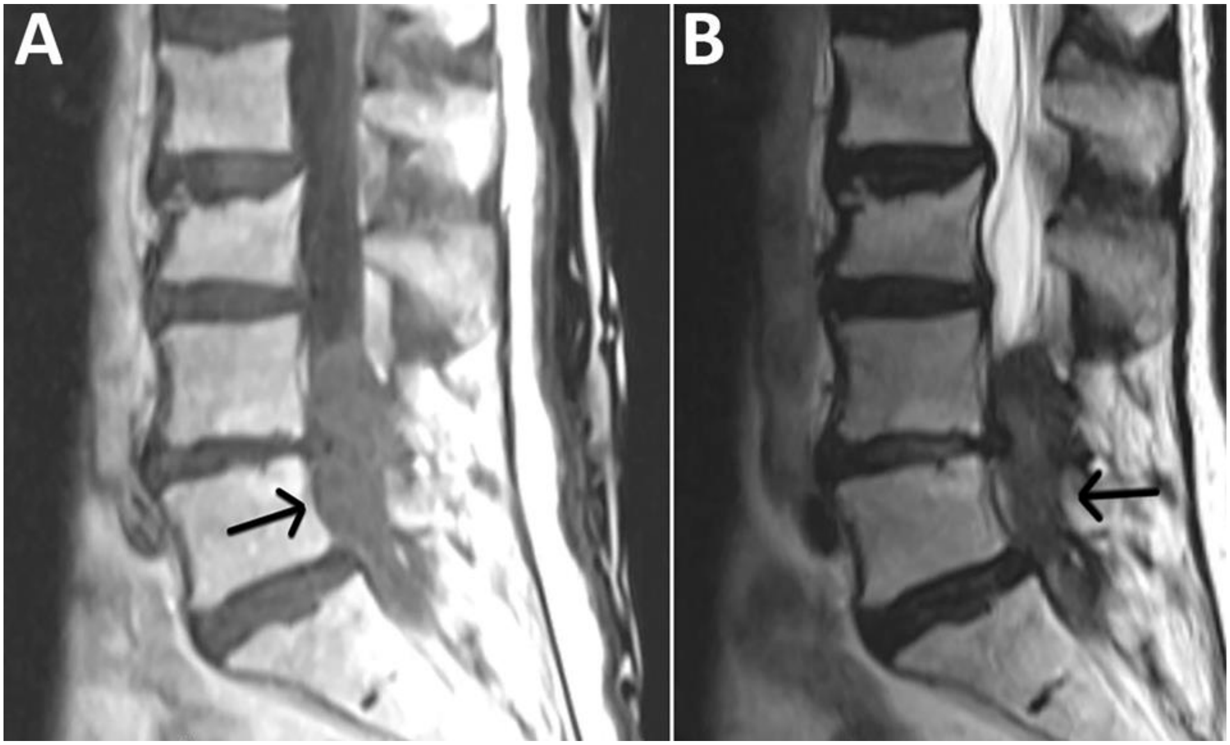

Technical Appendix Figure. A) Contrast-enhanced lumbar magnetic resonance image of T1 demonstrated a homogeneously enhancing intradural mass spanning from L4 to the sacrum. B) Corresponding T2 hypointense signal clearly demarking intradural location.
